# Anti-Inflammatory Pathways Modulated by Microbial Polysaccharides from Euganean Thermal Muds in Zebrafish

**DOI:** 10.3390/antiox14070878

**Published:** 2025-07-17

**Authors:** Micol Caichiolo, Raffaella Margherita Zampieri, Francesca Terrin, Annachiara Tesoriere, Fabrizio Caldara, Nicoletta La Rocca, Paolo Martini, Luisa Dalla Valle

**Affiliations:** 1 Department of Biology, University of Padova, Via U. Bassi 58/b, 35131 Padova, Italy; micol.caichiolo@phd.unipd.it (M.C.); raffaellamargherita.zampieri@unifi.it (R.M.Z.); francesca.terrin@phd.unipd.it (F.T.); annachiara.tesoriere@phd.unipd.it (A.T.); nicoletta.larocca@unipd.it (N.L.R.); 2 Pietro d’Abano Thermal Studies Center, Via Jappelli 5, 35031 Padova, Italy; fabrizio.caldara@centrostuditermali.org; 3 Department of Molecular and Translational Medicine, University of Brescia, Viale Europa 11, 25123 Brescia, Italy

**Keywords:** thermal mud, peloids, microbial polysaccharides, bioactive molecules, transcriptome, zebrafish, inflammation, copper sulphate

## Abstract

Thermal mud produced by spas of the Euganean Thermal District (Italy) has been used since ancient times for therapeutic purposes. Recently, the anti-inflammatory activity of microbial polysaccharides (M-PS), extracted from traditionally maturated muds, was demonstrated using the zebrafish model organism. However, the downstream signalling pathways regulated by M-PS remain largely unknown. In this study, to investigate the underlying mechanisms of inflammation resolution, we performed a transcriptome analysis on zebrafish larvae inflamed with copper sulphate and treated with M-PS. Our findings revealed that M-PS treatment down-regulated the expression of key genes involved in several inflammatory pathways. Gene Set Enrichment Analysis identified eleven up-regulated pathways (e.g., TNF-α signalling via NFκB, IL6–JAK–STAT signalling, p53 pathway, apoptosis, and interferon response) with components reduced in number and expression level in M-PS-treated larvae compared to the inflamed ones. Additionally, seven down-regulated pathways were identified (e.g., transcription factors E2F, MYC, and the G2M checkpoint). DEG-pseudotime analysis further confirmed the association of these genes with the pathways identified by GSEA. These results provide valuable insights into the anti-inflammatory properties of M-PS and the therapeutic potential of Euganean thermal muds for inflammatory diseases.

## 1. Introduction

Inflammation is a crucial body defence mechanism against harmful stimuli such as pathogenic microbes, damaged cells, toxic chemicals, or physical stressors. The process involves a complex network of cellular and molecular effectors of the immune system, working together to provide protection and to prevent irreversible damage. This process is tightly regulated to last only the time required to counter and eliminate the threat without causing excessive tissue damage. Any failure in the regulatory mechanisms delays resolution, leading to chronic inflammation and blocking the return to homeostasis. While acute inflammation is a crucial defence mechanism for well-being, chronic inflammation is detrimental and can cause permanent damage, compromise tissue integrity, and contribute to the pathogenesis of long-lasting inflammatory diseases [1].

Rheumatoid arthritis (RA) and related disorders are joint inflammatory diseases caused by autoimmune processes. Additionally, osteoarthritis (OA), one of the most common rheumatic disorders, long considered a non-inflammatory condition, is now assumed to be related to the presence of inflammation in the synovial tissue of patients [2].

Although different anti-inflammatory drugs are widely available and currently used, they often present harmful side effects that limit their long-term therapeutic potential. For this reason, there is a growing demand for the development of novel anti-inflammatory molecules or the validation of natural products [3].

In this context, pelotherapy, consisting of the external application of peloids (i.e., thermal muds) for therapeutic purposes, represents a well-established strategy to reduce perceived pain, improve mobility, and diminish inflammation associated with several pathologies such as RA, OA, osteoporosis, skin diseases, and other pathologic conditions [4,5]. For example, peloids (or therapeutic muds) of the Euganean Thermal Districts (Italy) are matured with hyperthermal water (>38 °C) and are recognized by the Italian health system as a therapeutic treatment for such pathologies. This effectiveness is attributed not only to the heat and electrolytes of the thermal water but also to the anti-inflammatory action of different bioactive molecules, such as glycoglycerolipids [6,7,8] and polysaccharides [9,10,11]. These molecules are produced by the complex microbial community that colonizes the mud during its maturation [12], a process strictly codified by regional guidelines [13].

Zebrafish is a model organism widely used to investigate the inflammatory process [14,15] and to analyse in vivo the properties of novel bioactive natural products [1,16]. In this regard, zebrafish was recently used to demonstrate the ability of polysaccharides extracted from traditionally matured therapeutic mud (hereafter referred to as M-PS, microbial polysaccharides) to ameliorate zebrafish inflammatory conditions [11]. In more detail, M-PS can counteract the inflammatory condition induced by either copper sulphate, UVB exposure, or tail fin amputation, as assessed in our previous works [9,10,11] by different experimental approaches such as analysis of specific morphometric traits and behavioural motor assays. Additionally, the reduction of luciferase signal in the transgenic zebrafish line Tg(8×Hs.NFκB:GFP, Luciferase), a reporter line for the NFκB transcription factor activity [17], was used as a readout of inflammation recovery. These findings were further supported by RT-qPCR analysis of genes involved in inflammatory pathways and in the oxidative stress response, two biological processes tightly interconnected and mutually dependent, thus confirming the efficacy of M-PS treatment [11].

The analysis of M-PS composition revealed the presence of galacturonic acid, mannose, xylose, ribose, and glucose, as well as a high percentage of sulphated groups in the polymers. These characteristics are all associated with the antioxidant and anti-inflammatory properties of polysaccharide molecules, as highlighted by Wang and collaborators [18].

Here, to gain a deeper understanding of the mechanisms of action and efficacy of these polysaccharides in resolving inflammatory conditions, we performed a transcriptome analysis on zebrafish larvae subjected to M-PS treatment, following chemical-induced inflammation. Ultimately, we aim to enhance scientific knowledge regarding the beneficial properties of Euganean thermal muds.

## 2. Materials and Methods

### 2.1. Microbial Polysaccharides

The M-PS (microbial polysaccharides) used in this study were the same as in Zampieri et al., 2022 [11]. Consequently, the reader can refer to that paper for a description of the method used for polysaccharides’ extraction from therapeutic mud samples, the chemical analyses performed (monosaccharide composition, sulphate groups abundance, and FT-IR spectrum) and the final chemical composition of these molecules. Lyophilised M-PS samples were stored in a fresh and dark cabinet before their use. For zebrafish larvae experiments, M-PS were weighed and dissolved in fish water (FW) at the concentration of 50 µg/mL.

### 2.2. Zebrafish Maintenance

Zebrafish were maintained following standard guidelines, as well as fish breeding for egg spawning, collection, and staging [19]. Embryos were raised in fish water in petri dishes at 28 °C. Embryonic and larval stages were reported as hours or days post fertilization (hpf, dpf). No adult zebrafish were sacrificed for this study. All experiments were performed on larvae before the free-feeding stage and thus did not fall under animal experimentation laws according to EU Animal Protection Directive 2010/63/EU.

### 2.3. Zebrafish Larvae Treatment

Chemical treatment with CuSO_4_∙5 H_2_O (1027841000, Merck, Darmstadt, Germany) was performed using the bath immersion method on 3-dpf larvae of the same reproduction for each biological replicate. Each of the five experimental condition groups consisted of 15–20 individuals. The five experimental conditions were: (1) larvae kept in FW and used as the control condition (hereafter indicated as “CTRL”); (2) larvae exposed to M-PS for 4 h (“M-PS” treatment); (3–5) three groups of larvae exposed to 20 µM of CuSO_4_∙5 H_2_O for 2 h. After the inflammatory induction, larvae were carefully rinsed with FW (four times for 15 min) to remove any residual trace of the chemical. Then, one group (3) was given only FW (hereafter indicated as “INF”), one group (4) was subsequently exposed to M-PS for 2 h (hereafter indicated as “INF + 2 h” treatment), and one group (5) was exposed to M-PS for 4 h (hereafter indicated as “INF + 4 h” treatment). At the end of each treatment, total RNA was extracted. Larvae were euthanized after 7 h from the beginning of the experiment, except for the INF + 2 h treatment, for which RNA was obtained after 5 h (2 h of inflammation, 1 h washing, and 2 h of M-PS exposure). A schematic representation of the treatments is reported in Figure S1A. The experiment was performed using 4 biological replicates.

### 2.4. Library Construction and High-Throughput Sequencing

Total RNA was extracted from larvae using TRIzol (15596018, Thermo Fisher Scientific, Milan, Italy). Samples were resuspended in 10 µL of RNase-free water and quantified using a Nanodrop (Thermo Fisher Scientific, Waltham, MA, USA). The quality was assessed with an RNA Bioanalyzer (Agilent, Santa Clara, CA, USA), obtaining for all samples RNA Integrity Numbers (RIN) > 8. Sequencing of 2 μg of RNA was performed on a NextSeq500 ILLUMINA to produce at least 10 million reads (75 bp SE) per sample. Quant Seq 3′ mRNA-seq Library Prep kit (Lexogen GmbH, Vienna, Austria) was used for library construction. The sequencing and library construction were performed by the NGS Sequencing Core of the Biology Department of the University of Padova.

### 2.5. Processing of RNA-Seq Data and Bioinformatics Analysis

Transcript quantification was performed using Salmon (v1.9.0) [20] with transcripts defined in Ensembl 104 (GRCz11). Quantification analysis revealed an average of 10.5 M counts per sample (min 8.5 M; max 13.5 M). Genes with more than 10 counts in at least 4 samples were retained for further analysis. Gene expression levels were estimated with the tximport R package (v1.30.0) [21].

PCA was performed using the prcomp R function on the top 500 variant genes extracted from the expression matrix (normalized by variance stabilizing transformation from the DESeq2 R package v1.42.1), which was corrected for batch effect with the ComBat_seq function from the sva R package (v3.50.0).

Differentially expressed genes were computed using the DESeq2 R package (v1.42.1) [22]. Following DESeq2 best practices, differentially expressed genes were computed using raw counts, including the batch effect in the model.

Time activation analysis was performed using the degPatterns function from the DEGreport R package (v 1.38.5) [23] on rlog (DESeq2) normalized expression data. Pseudo-time analysis was performed to detect patterns of expression by considering conditions as sequential time events (DESeq2 likelihood ratio test + DEGreport).

Enrichment analysis was carried out using the clusterProfiler R package (v4.10.1) [24]. To understand the main regulatory pathways that were targeted by the M-PS treatment, we performed enrichment analysis using the Hallmark gene set from the Molecular Signature Database (GSEA). Pathway annotations were downloaded from the msigdbr r package (v7.5.1).

All analyses were performed using R v4.3.2 unless otherwise stated.

### 2.6. Real-Time Quantitative PCR (RT-qPCR)

For the validation of the RNAseq results, 4 new zebrafish larvae replicates were prepared. With respect to the experimental conditions used for transcriptome analysis, no larvae only treated with M-PS were used. Moreover, after inflammatory induction (prolonged to 2.5 h), larvae were washed and then treated for 4 and 6 h (hereafter indicated as “INF + 4 h” and “INF + 6 h”).

At the end of each treatment, total RNA was extracted: larvae were euthanized after 9.5 h from the beginning of the experiment, except for the INF + 4 h treatment, for which RNA was obtained after 7.5 h (2.5 h of inflammation, 1 h of washing, and 4 h of M-PS exposure). A schematic representation of the treatments is reported in Figure S1B.

For expression analysis, total RNA was extracted from pools of 15–20 3-dpf larvae with TRIzol reagent. Poly(A) mRNA was purified from 5 μg of total RNA with Dynabeads “mRNA direct kit” (61011, Thermo Fisher Scientific, Milan, Italy) and used for cDNA synthesis with the High-Capacity cDNA Reverse Transcription Kit (4368813, Thermo Fisher Scientific, Milan, Italy) according to the manufacturer’s protocol. Real-time qPCR was performed in triplicate with the SYBR green method using the CFX384 Touch Real-Time PCR Detection System (Bio-Rad Laboratories, Hercules, CA, USA) and the 5 × HOT FIREpol EvaGreen qPCR Mix Plus (08-24-0000S, Solis BioDyne, Tartu, Estonia). Data were normalized for the expression of *glyceraldehyde-3-phosphate dehydrogenase* (*gapdh*) housekeeping gene to account for the initial concentration and quality of the total RNA. The amplification protocol was 95 °C for 12 min, followed by 40 cycles at 95 °C for 30 s, 60 °C for 30 s, and 72 °C for 30 s. No amplification products were observed in negative controls, and no primer–dimer formations in the control templates. Results were analysed according to the ΔΔCt method using the Bio-Rad CFX Manager software Version 3.1 (Bio-Rad Laboratories, Hercules, CA, USA). Four biological replicates of the experiments were performed, and all reactions were done as technical triplicates. Each primer pair was designed to span an intron, except for the *hsp70.3* gene, which does not contain introns. Primer sequences are reported in Table S1. The identity of the amplified fragment was analysed by sequencing. Primer efficiency was calculated and accounted for in the calculation of ΔΔCt.

Experiments were conducted at least three times with biological replicates composed of 15–20 larvae.

### 2.7. Acridine Orange Staining

Apoptosis analysis in the whole body of zebrafish larvae was conducted using acridine orange (AO) staining. To induce inflammation, 3-dpf larvae were exposed to 20 µM copper sulphate for two hours and then incubated in either FW or 50 µg/mL M-PS solution (samples named “INF” and “INF + 2 h”) for an additional two hours. Larvae exposed only to FW served as controls (named “CTRL”).

At the end of the treatments, larvae were rinsed with FW and immersed in 5 µg/mL acridine orange (A6014, Merk, Darmstadt, Germany) solution for 20 min. Larvae were then rinsed 2–3 times with FW, anesthetized with tricaine, and positioned sideways in 2.5% methylcellulose on microscope slides.

Fluorescent images were captured using a Leica M165 FC stereoscopic microscope equipped with a Leica DFC7000 T digital camera (Leica Microsystems, Milan, Italy). The areas of apoptotic cells with fluorescent AO positivity were quantified using ImageJ (v2.14.0) to compare treated larvae with controls. Different regions of interest (ROIs) were defined: the whole larva, the yolk area, and the trunk area (posterior to the anus). RawIntDen values were measured for each ROI. A schematic representation of the measurement process is shown in Figure S2. The final results for the whole larva were obtained by subtracting the RawIntDen values of the yolk area, whereas trunk RawIntDen values were used directly, as represented in paragraph 3.5.

Experiments were conducted three times with replicates composed of 10–12 larvae obtained from distinct spawns.

### 2.8. Total Antioxidant Capacity (TAC) Assay

The total antioxidant capacity (TAC) was established using a commercial kit (CAK1111, CliniSciences, Guidonia Montecelio, Italy) following the manufacturer’s protocol. Specifically, about 30 3-dpf larvae, exposed for 4 or 6 h to either M-PS (INF + 4 h or INF + 6 h) or FW (INF) after the inflammation with copper sulphate, were homogenized in 250 μL of the commercial assay buffer using a sonicator (3 × 10 s) in ice and then incubated for 10 min in ice before the centrifugation step. The quantification of proteins was carried out using the Pierce BCA Protein Assay Kit following the manufacturer’s instructions (23225, Thermo Fisher Scientific, Milan, Italy).

The results were calculated according to the equation of the line y = 0.3218x + 0.0181, where y is the absorbance and x is the concentration (R^2^ = 0.9991) after a 15 min incubation. The analysis was carried out using five biological replicates, with results expressed in U/mg according to the protein concentration of the sample.

### 2.9. Statistical Analysis for the Validation Experiments

Statistical analysis was performed using GraphPad Prism (Version 10.2.3, GraphPad software). Given that all the comparisons included more than two groups, a one-way ANOVA was conducted. When the assumption of equal variances was met, Tukey’s multiple comparisons test was used for post hoc analysis. Instead, when group variances were not homogeneous (as assessed by GraphPad’s built-in variance tests), we applied Brown–Forsythe and Welch one-way ANOVA to account for heteroscedasticity. Here, Dunnett’s T3 multiple comparisons test, which does not assume equal variances and calculates individual variances for each comparison, was used as the post hoc test. This ensures a more accurate evaluation of group differences under conditions of unequal variance. Significance was set at *p* < 0.05, and *p*-values were indicated either with: * *p* < 0.05, ** *p* < 0.01, *** *p* < 0.001, **** *p* < 0.0001, or with letters. In this case, the exact adjusted *p*-values are provided in the Supplementary Materials. Data are presented as means ± SD, and the specific test used is indicated in the figure captions.

## 3. Results

### 3.1. Identification of Differentially Expressed Genes (DEGs) Following Copper Sulphate Exposure and M-PS Treatment

In our previous paper, we investigated the composition of M-PS, extracted from peloids produced according to the codified mud maturation protocol by a spa of the Euganean Thermal District, and assessed their beneficial properties using the zebrafish model [11]. Briefly, the monosaccharide composition revealed a predominance of galacturonic acid, mannose, xylose, ribose, and glucose, indicating that M-PSs are highly negatively charged and hydrophilic polymers [11]. Notably, the sulphate group content, as confirmed by FT-IR spectral analysis, was found to be very high, reaching 13.62% *w*/*w* (±1.48), a feature already reported for polysaccharides with tested anti-inflammatory [25] and antioxidant activities [26].

To gain a deeper understanding of the anti-inflammatory effects obtained with M-PS treatment, we performed a transcriptomic analysis of samples obtained with a similar experimental approach, as illustrated in Figure S1A. In particular, after zebrafish inflammation induction with copper sulphate (CuSO_4_) (INF), 3-dpf larvae were treated with M-PS for two time intervals (INF + 2 h, INF + 4 h).

**Figure 1:**
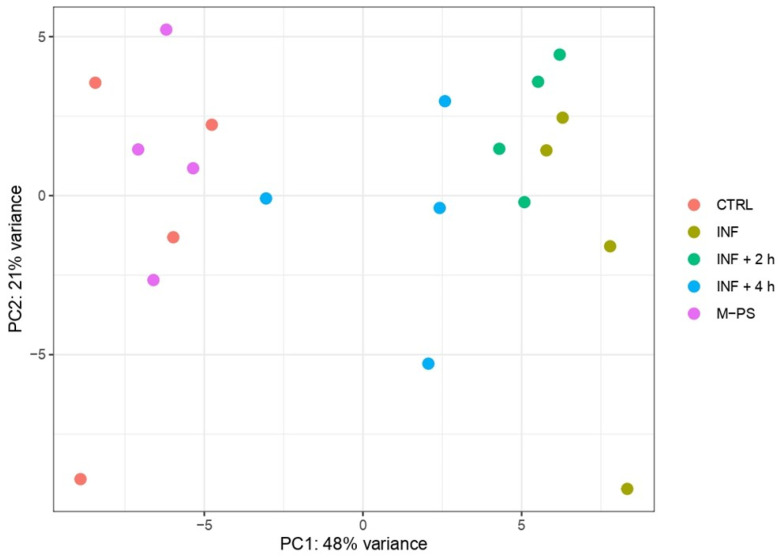
Principal component analysis of top 500 most variant genes after batch correction. The first (PC1) and second principal components (PC2) are shown on the horizontal and vertical axes, respectively.

Principal component analysis (PCA) revealed that PC1 accounts for the transcriptional differences triggered by the treatment applied to the larvae. Specifically, controls and larvae treated only with CuSO_4_ (inflamed larvae, INF) present the most divergent transcriptomes, while larvae treated with M-PS after the inflammatory induction display intermediate transcriptional states, with larvae treated for 4 h deviating more from inflamed samples. This suggested a progressive attenuation of the inflammatory transcriptional state as the M-PS treatment proceeds over time (Figure 1).

Samples treated only with polysaccharides showed no differences and clustered with untreated larvae samples, confirming the lack of toxicity or adverse effects of the bioactive molecules used [10]. The PCA result indicated that the inflammatory status is the main factor explaining the expression profiles of the samples. The dispersion of the points belonging to the same treatment groups observed in PC2 (accounting for 21% of the variance) is likely due to the variability among different larval batches.

To gain more insight into the transcriptional differences, we performed differential expression analysis using controls (CTRL) as the reference condition. We retrieved 1457 differentially expressed genes (DEGs; adjusted *p*-value ≤ 0.05) between INF larvae and CTRL (694 up- and 763 down-regulated). The analysis revealed a gradual decrease in the number of DEGs in larvae treated with M-PS after the inflammatory induction. In fact, we obtained 1137 DEGs between CTRL and INF + 2 h larvae (552 up- and 585 down-regulated) and 299 DEGs between CTRL and INF + 4 h larvae (124 up- and 175 down-regulated). As expected from the PCA analysis, the treatment with only M-PS had almost no effects (19 DEGs in M-PS versus CTRL larvae; 6 up- and 13 down-regulated).

### 3.2. Gene Set Enrichment Analysis (GSEA) and Functional Classification of the Differentially Expressed Genes

We used GSEA to focus on relevant biological pathways activated or modulated by the inflammatory induction, rather than on individual gene alterations. Specifically, we could verify if the M-PS treatment has anti-inflammatory functions and how it contributes to the recovery from the pathological condition. A summary of all the significant hallmark gene sets obtained by the analysis of the up- and down-regulated genes (adjusted *p*-value ≤ 0.1) is reported in Tables S2 and S3, respectively.

**Figure 2:**
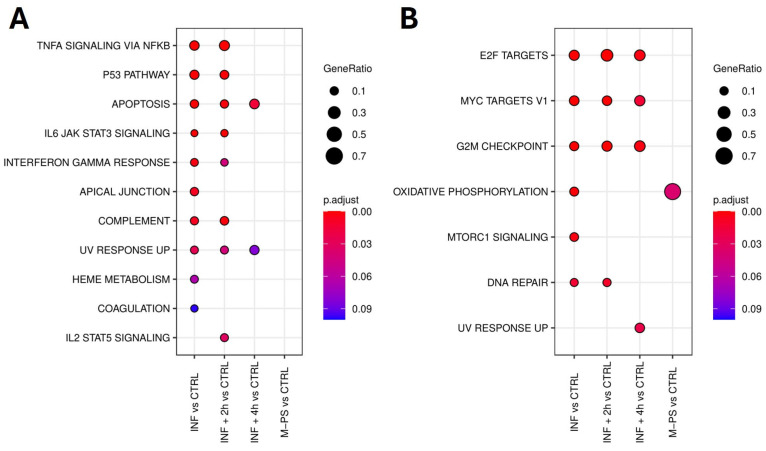
Top 10 enriched hallmark gene sets in each condition (adjusted *p*-value ≤ 0.1) identified from the analysis of up-regulated genes. No DEGs mapped to hallmark gene sets, and no enriched hallmarks were found for the M-PS vs. CTRL comparison (**A**). Top 10 enriched hallmark gene sets in each comparison (adjusted *p*-value ≤ 0.1) identified from the analysis of down-regulated genes (**B**).

Firstly, we analysed the up-regulated genes by comparing them across our different conditions. We found 11 pathways that were activated upon inflammation with copper sulphate. All of them were relevant for inflammation and mainly refer to TNF-α signalling via NFκB, IL6-JAK-STAT signalling, p53 pathway, and apoptosis. After a 2-h M-PS treatment, we observed a reduced number of up-regulated genes in some of these pathways (Figure 2A). However, following a 4-h M-PS treatment, most of these pathways no longer contained significantly up-regulated genes, meaning that nearly all the molecular circuits activated in response to copper sulphate have returned to baseline levels. Treatment of zebrafish larvae with M-PS alone did not result in any up-regulated genes, confirming M-PS’s lack of toxicological effects (Figure 2A), as already demonstrated with human cells treated with polysaccharides extracted from cyanobacteria [10].

Similarly, we analysed the down-regulated subset of genes. Overall, we observed that, among the molecular pathways switched off by the copper sulphate treatment, three of them remained down-regulated also in the INF + 4 h group, although reduced in gene number and in the extent of down-regulation (Figure 2B). These are the enriched hallmark genes corresponding to the transcription factors E2F, MYC, and the G2M checkpoint.

**Figure 3:**
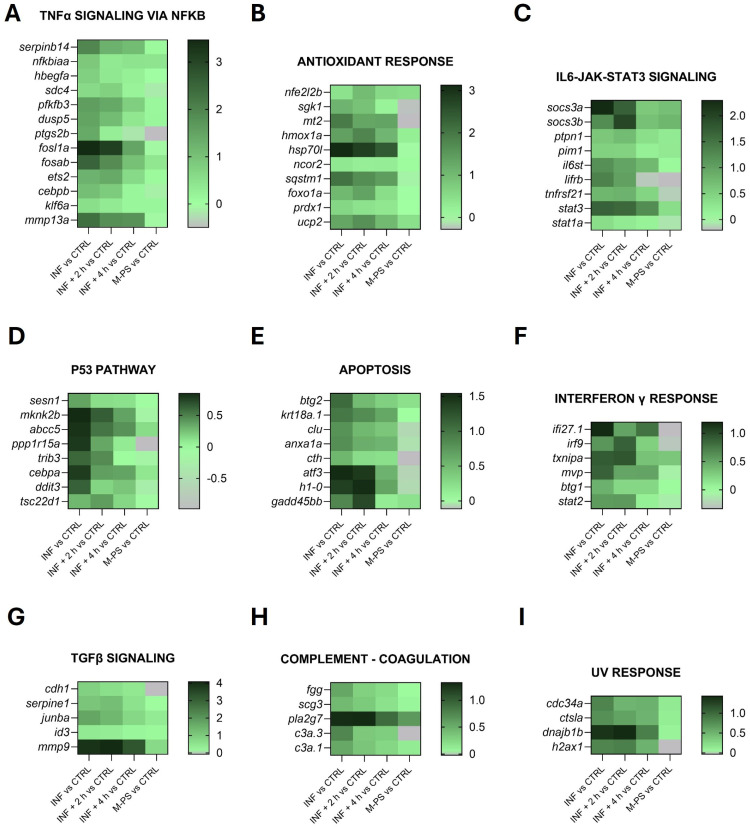
Heatmaps of genes identified in pathways up-regulated by CuSO_4_⋅5 H_2_O exposure (INF). The pathways have been selected from the ones listed in Figure 2A and Table S2, as well as specifically selected from the DEGs as genes related to the antioxidant response (graph B). Selected representative genes attributed to: (**A**) TNFα signalling via NFκB pathway; (**B**) antioxidant response pathway; (**C**) IL6-JAK-STAT3 pathway; (**D**) p53 pathway; (**E**) apoptosis pathway; (**F**) interferon γ response pathway; (**G**) TGFβ signalling pathway; (**H**) coagulation pathway; (**I**) UV response pathway. The graphs were created with GraphPad Prism 10; the legend represents the log_2_Foldchange of each treatment compared to the control (CTRL) condition (mean of the four biological replicates).

Figure 3 shows the heatmaps of some representative up-regulated genes belonging to the principal pathways identified by the GSEA analysis in Figure 2A.

As reported by Pereira and collaborators [27], copper sulphate induces inflammation primarily through the generation of oxidative stress, acting as a pro-oxidant metal that catalyses the production of reactive oxygen species (ROS) via redox cycling reactions.

In agreement with this, although the genes identified by the GSEA analysis (Figure 2A) are mainly involved in inflammatory response pathways (Figure 3), we also identified additional genes related to the antioxidant response from the list of differentially expressed genes (some of these are reported in Figure 3B).

**Figure 4:**
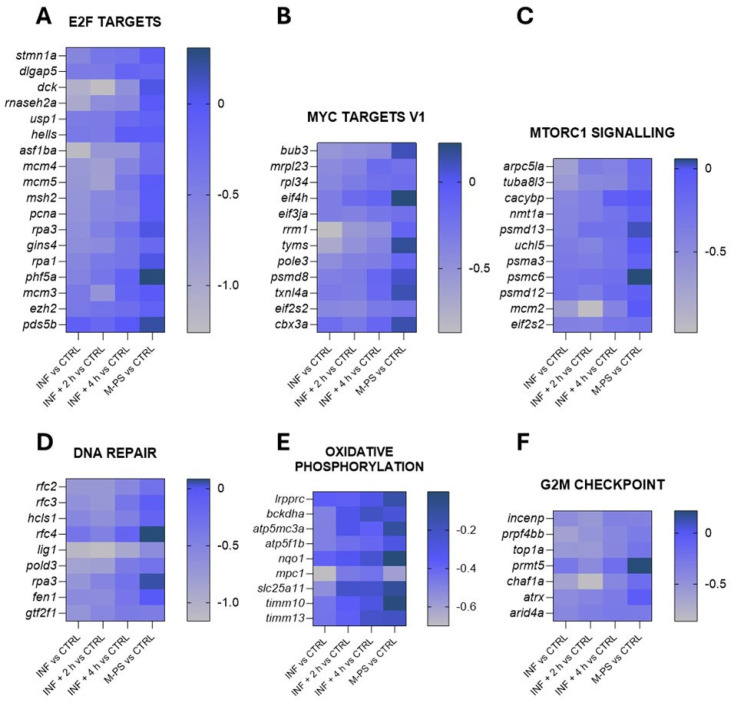
Heatmaps of genes identified in pathways down-regulated by CuSO_4_⋅5 H_2_O exposure (INF). The pathways have been selected from the ones listed in Figure 2B and Table S3. Selected representative genes attributed to: (**A**) E2F targets pathway; (**B**) MYC targets V1 pathway; (**C**) MTORC1 signalling pathway; (**D**) DNA repair pathway; (**E**) oxidative phosphorylation pathway; (**F**) G2M checkpoint pathway. The graphs were created with GraphPad Prism 10; the legend represents the log_2_Foldchange of each treatment compared to the control (CTRL) condition (mean of the four biological replicates).

The down-regulated genes, as shown in Figure 2B, were instead mainly associated with key regulators of the cell cycle, such as transcription factors E2F, MYC targets, the G2M checkpoint, and DNA repair. Some of the genes belonging to these pathways are reported in Figure 4.

These pathways are highly interconnected and crucial for the regulation of cell homeostasis, proliferation, and genomic integrity. Their down-regulation could partially be due to the up-regulation of genes of the p53 and apoptosis pathways, which were induced by the inflammatory status (Figure 3C,H).

Interestingly, even after a 4-h M-PS treatment, the transcriptional levels of some of these down-regulated genes did not reach those of controls.

Finally, GO enrichment analysis of DEGs was also performed, again comparing each treated group against the control group. The results of this analysis, with the list of all the genes altered in biological process (BP), cellular component (CC), and molecular function (MF), are reported on the online repository Zenodo (https://doi.org/10.5281/zenodo.13843249, accessed on 26 September 2024).

### 3.3. DEG-Pseudotime Patterns Analysis of the Differentially Expressed Genes

To exclude that the absence of inflammation-related pathways/genes in the “4-h M-PS-treatment” group was not due to differences in DEG numbers, we performed a pseudo-temporal analysis by considering conditions as sequential time points. We chose to limit our temporal pattern search among the DEGs obtained from the inflamed larvae *versus* control, to observe the expression evolution of genes that were specifically altered by inflammation.

**Figure 5:**
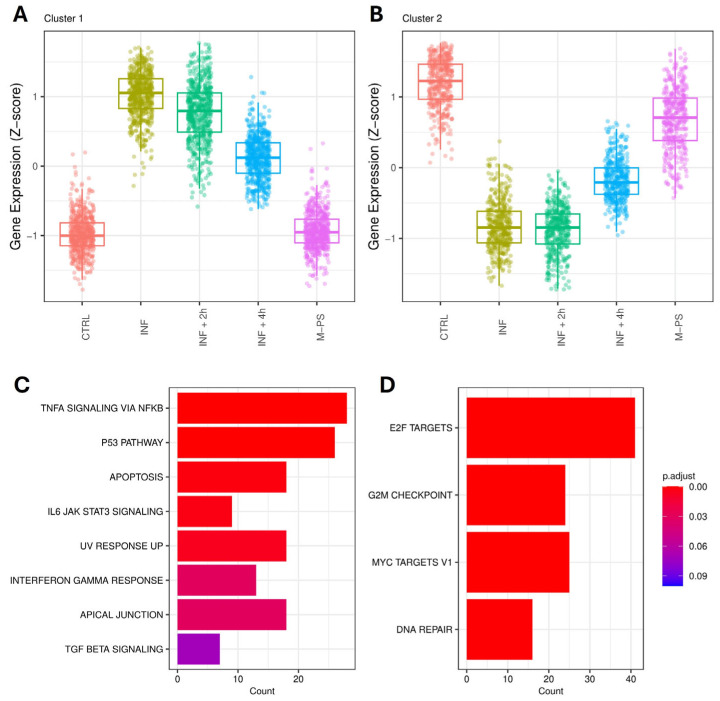
Pseudo-temporal patterns of genes altered by inflammation. The expression of the 573 genes in cluster 1 (**A**) and 415 genes in cluster 2 (**B**) is shown across the five conditions, temporally ordered as follows: control (CTRL), inflammation (INF5), 2-h M-PS treatment following inflammation (INF + 2 h), 4-h M-PS treatment following inflammation (INF + 4 h), and M-PS treatment only. Top 10 hallmark gene sets (adjusted *p*-value ≤ 0.1) were obtained from the enrichment analysis for “activated” genes, those belonging to cluster 1 (**C**), and “repressed” genes, those in cluster 2 (**D**).

Pseudo-temporal pattern analysis identified fourteen clusters (three were discarded due to low gene number) (Figure S3). We focused on the larger clusters to observe the strongest signals. Cluster 1 contains 573 genes with a clear activation pattern (hereafter referred to as “activated” genes). Activated genes have an expression profile that starts from the basal level of the controls, peaks with the copper sulphate treatment, and then gradually decreases after the 2- and the 4-h treatment with M-PS (Figure 5A).

Cluster 2 contains 415 genes with a repression pattern (hereafter referred to as “repressed” genes). These genes are down-regulated after the copper sulphate treatment and then gradually return to basal levels after the 4-h M-PS treatment (Figure 5B). Both “activated” and “repressed” genes remain substantially unaffected by M-PS treatment alone.

We performed enrichment analysis on the activated and repressed genes and confirmed that up- and down-regulated genes refer to pathways modulated by inflammation with copper sulphate and then de-activated by the treatment with M-PS, like in the previous GSEA analysis.

In particular, we can highlight the presence of TNFα signalling via NFκB, IL6-JAK-STAT signalling, and Interferon gamma response, whose genes are activated and then their expression is brought back to levels similar to those in the control samples (Figure 5C).

Repressed genes are enriched for gene sets related to the cell cycle, MYC, and E2F (Figure 5D).

### 3.4. Validation of Transcriptomic Analysis by RT-qPCR

The results obtained from the transcriptomic analysis were validated through RT-qPCR performed on four new replicates obtained with the same experimental approach used for RNAseq. To further confirm the potential of M-PS in reducing the transcription of genes of the inflammatory pathways, we extended the inflammation exposure to 2.5 h and evaluated the recovery of the inflammatory status after 4 h (INF + 4 h) and 6 h (INF + 6 h) of M-PS treatment (Figure S1B).

**Figure 6:**
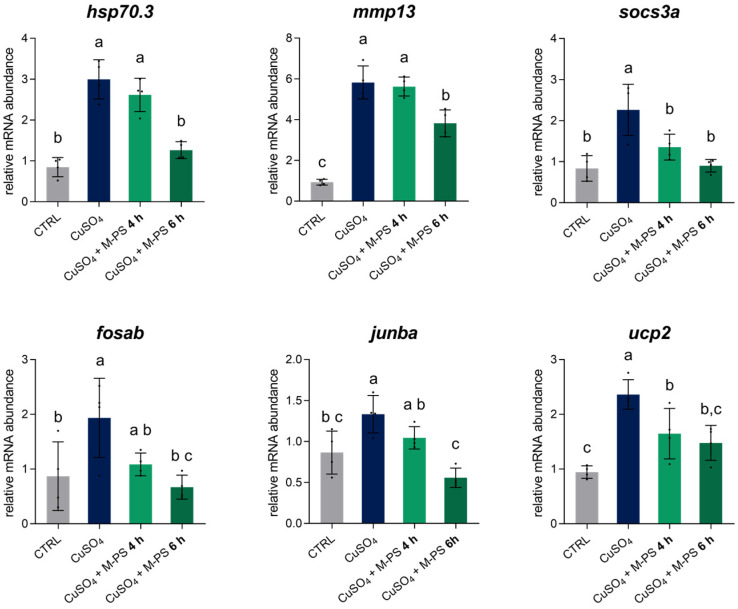
Relative mRNA abundance of genes involved in the inflammatory response, analysed in 3-dpf zebrafish larvae after CuSO_4_⋅5 H_2_O exposure (INF), with 4 or 6 h of M-PS treatment (INF + 4 h, INF + 6 h). The bars represent mean ± SD of four independent experiments conducted with 15–20 larvae. Statistical analysis was performed using GraphPad Prism 10 (ordinary one-way ANOVA followed by Tukey’s multiple comparisons test with a single pooled variance). Statistical significance was set at *p* < 0.05, and the results of the multiple comparisons are shown with letters (different letters show differences among data). The exact adjusted *p*-values are listed in Table S4.

As shown in Figure 6, we analysed six genes: *hsp70.3*, *mmp13*, *socs3a*, *fosab*, *junba*, and *ucp2*. These genes were all up-regulated following copper sulphate-induced inflammation and subsequently down-regulated after M-PS treatment. Moreover, as treatment progresses, the expression level gradually approaches the values of the control samples.

In particular, we analysed the transcription of the *hsp70.3* gene, encoding for a member of the heat shock protein family Hsp70, whose up-regulation after cellular stress is linked to its protective and immunomodulatory role in recovering cell homeostasis [28]. After six hours of M-PS treatment, the expression level of this gene returned to the basal level, supporting the role of M-PS in the effective resolution from the inflammatory condition. We also validated the *mmp13* gene, which plays a key role in matrix degradation and tissue remodelling during inflammation, and that is also normally up-regulated in OA and RA [29,30]. The down-regulation of this gene, as obtained from both RNA-seq analysis and RT-qPCR, is consistent with previous studies involving this particular M-PS [11], as well as with exopolysaccharides extracted directly from the cyanobacterium *Phormidium* sp. ETS-05 and used on zebrafish larvae inflamed by tail fin amputation [10]. Moreover, we validated *socs3a*, a paralog of *socs3b*, and *fosab* and *junba*, two components of the complex AP-1, whose transcriptional activity is linked to the immune response [31]. Finally, we analysed the expression of the *ucp2* gene, encoding the *uncoupling protein 2*, which is activated by ROS and works to maintain the redox balance inside mitochondria and to contribute to the antioxidant defence [32]. This gene was also up-regulated by the induction of inflammation and finally returned to control levels with M-PS treatment.

### 3.5. Validation of the Recovery from Increased Apoptosis Levels Induced by Copper Sulphate

To better characterize the beneficial properties of the M-PS, and indirectly of the muds used for the therapeutic treatments, we decided to analyse in vivo two correlated pathways that emerged from the transcriptomic analysis and were not taken into consideration in our previous publications.

**Figure 7:**
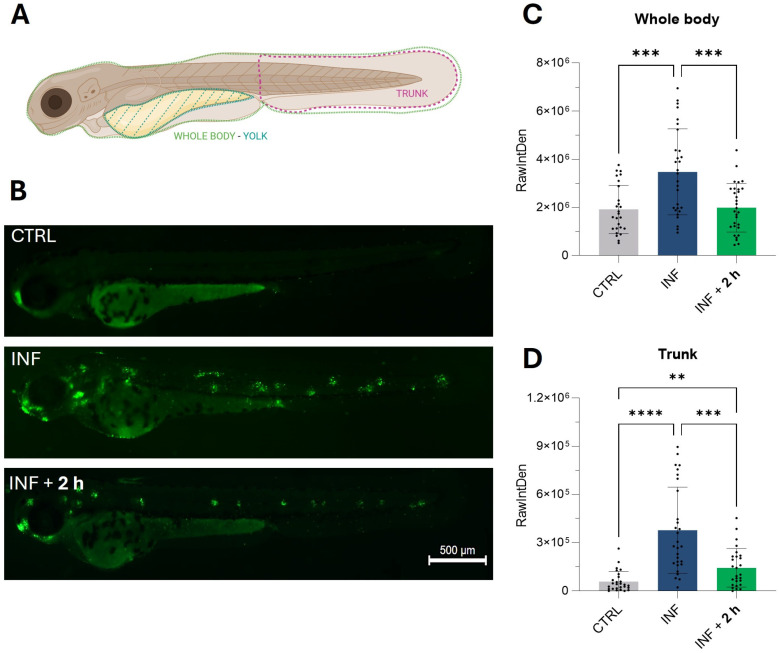
Results of the acridine orange staining on 3-dpf zebrafish larvae two hours after CuSO_4_⋅5 H_2_O exposure (INF) and two hours after M-PS exposure (INF + 2 h). (**A**) Schematic representation of the areas considered for the measurements of RawIntDen. Whole-body results were obtained by subtracting the RawIntDen of the yolk from that of the whole larvae. Image created with Biorender.com. (**B**) Micrographs showing augmented apoptosis due to CuSO_4_⋅ 5 H_2_O exposure (INF) and rescue of neuromasts with M-PS 50 μg/mL treatment (INF + 2 h). Sibling larvae (CTRL) are shown for comparison. Scale bar = 500 µm. Recovery of apoptosis level in the whole body (**C**) and trunk (**D**). The explanation of RawIntDen measurement is reported in Figure S2. The bars represent mean ± SD of three independent experiments conducted with 10–12 larvae per treatment. Statistical analysis was performed using GraphPad Prism 10 (Brown–Forsythe and Welch ANOVA test followed by Dunnett’s T3 multiple comparisons test with individual variances computed for each comparison). Statistical significance was set at *p* < 0.05, and the results of the multiple comparisons are shown with: ** *p* < 0.01, *** *p* < 0.001, **** *p* < 0.0001.

As shown in the heatmaps (Figure 3D and Figure 3E, respectively), genes from the p53 and apoptosis pathways were up-regulated by inflammation and subsequently down-regulated following exposure to M-PS. Therefore, we visualized the apoptotic response using acridine orange, a dye capable of permeating dying cells and commonly used to detect apoptosis [33]. It is known that copper sulphate can rapidly induce cell death, particularly in neuromasts distributed along the lateral line system of zebrafish larvae [34], as visible in the central fluorescence image of Figure 7B, showing death of mechanosensory hair cells. The results obtained after M-PS treatment showed a significant decrease in the level of apoptosis, both when analysing the whole body (Figure 7C) or the trunk region (Figure 7D).

### 3.6. Validation of the Recovery from the Oxidative Stress Induced by Copper Sulphate

In our previous work, we analysed ROS levels following copper sulphate exposure and detected a reduction in ROS levels after treatment with M-PS, using the DCFH-DA fluorescent probe [11]. In agreement with this, in the transcriptomic analysis presented here, we identified several genes related to oxidative stress that were up-regulated at early time points but returned to control levels after 4 h of M-PS treatment (Figure 3B). This pattern is common among antioxidant-related genes whose expression increases in response to oxidative stress to recover cell homeostasis.

**Figure 8:**
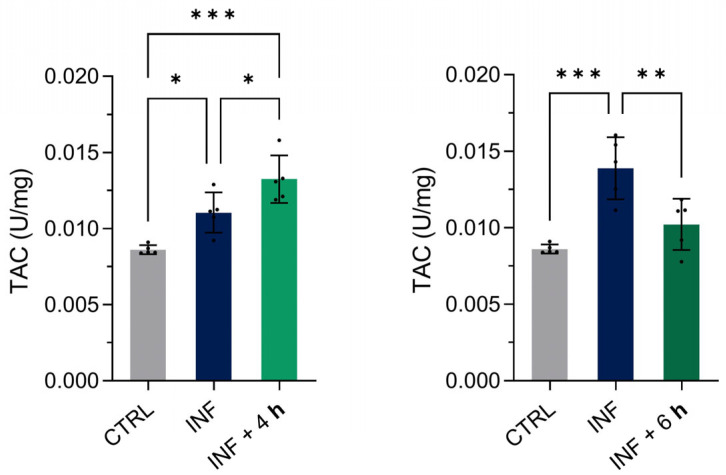
Results of the total antioxidant capacity (TAC) assay on 3-dpf zebrafish larvae two hours after CuSO_4_⋅5 H_2_O exposure (INF) and four or six hours after M-PS exposure (INF + 4 h on the left and INF + 6 h on the right, respectively). The results are expressed as U/mg of protein. The bars represent mean ± SD of five independent experiments conducted with 30 larvae per treatment. Statistical analysis was performed using GraphPad Prism 10 (ordinary one-way ANOVA test followed by Tukey’s multiple comparisons test). Statistical significance was set at *p* < 0.05, and the results of the multiple comparisons are shown with: * *p* < 0.05, ** *p* < 0.01, *** *p* < 0.001.

To further characterize the antioxidant response, we evaluated the total antioxidant capacity (TAC) of homogenized zebrafish larvae exposed to inflammation and subsequently treated with M-PS for 4 or 6 h (Figure 8). Compared to their only-inflamed siblings (INF), we observed an increase in TAC after 4 h and a decrease after 6 h of M-PS treatment. A gradual increase in TAC was observed in inflamed larvae from 4 to 6 h, suggesting persistent oxidative stress driven by persistently elevated ROS levels.

## 4. Discussion

The anti-inflammatory and antioxidant potential of natural polysaccharides has been extensively studied in the last few years, as reported by numerous reviews on this subject [35,36,37]. These molecules, which can derive from fungi, plants, and algae, have been shown to modulate the immune response and to affect different signalling pathways, thus regulating the expression and production of pro- and anti-inflammatory cytokines or counteracting oxidative stress [35,36,37]. Their study can lead to the use of polysaccharides as natural bioactive molecules in different applications ranging from functional food, pharmaceutical purposes, and cosmetics production [38].

The potential and effectiveness of thermal muds used for pelotherapy, a non-pharmacological therapeutic approach for rheumatic and osteoarticular diseases, have long been known and validated by clinical studies on patients, as reviewed in various papers [39,40,41].

However, the identification of the bioactive molecules produced by the microbiota, which progressively colonises the thermal mud during its maturation, and the characterization of their potential are still in their early stages.

In this regard, in recent years, we analysed the anti-inflammatory and antioxidant potential of polysaccharides produced by the mud microbiota to provide a scientific basis for the application of Euganean thermal muds [9,10,11].

In this study, we used RNA-seq combined with GSEA and the DEG-pseudotime pattern approach to conduct a comprehensive analysis of gene expression patterns in M-PS-treated zebrafish samples after copper sulphate-induced oxidative stress and inflammation. GSEA results revealed that these molecules counteract inflammation by reducing both the expression levels and the number of key genes involved in different pathways linked to stress and inflammation (e.g., TNF-α signalling via NFκB, IL6–JAK–STAT signalling, p53 pathway, apoptosis and interferon response).

While most of these genes showed the highest expression in the inflamed condition and a decrease with the progression of the treatment, some presented similar or even higher expression levels after two hours of M-PS treatment. This specific behaviour is likely related to their involvement in the inflammatory response together with protective activities such as tissue repair and regeneration. Thus, their expression at high levels can persist during the initial phase of M-PS treatment.

For example, *atf3*, a stress-inducible gene, can function as an apoptosis regulator [42] and as a transcriptional repressor, blocking the transcription of pro-inflammatory genes and thereby contributing to the resolution of inflammation [43]. Similarly, the increased expression of *socs3b* after two hours of M-PS treatment could be attributed to the protein’s role as a negative regulator of cytokine signalling [44].

Moreover, the increased expression of genes related to the antioxidant response, like *nfe2l2b*, *hmox1a,* and *ucp2,* is due to their specific roles in restoring cellular homeostasis [45,46,47]. In all cases, the majority of the up-regulated genes presented a clearly reduced expression after the extended M-PS treatment, ultimately reaching values comparable to the controls.

The activation of genes involved in inflammation-related pathways, along with the progressive modulation in the number of DEGs or in their expression levels following M-PS treatments, underscores the capability of these bioactive molecules to interact with several cellular molecular targets or to activate signal cascades that influence, both directly and indirectly, multiple signalling pathways.

Notably, the DEG-pseudotime pattern analysis confirmed that these genes are linked to the same pathways identified through GSEA, further emphasizing the close relationship between oxidative stress and inflammation resolution and exposure to these bioactive compounds.

Additionally, M-PS treatment partially recovered cell homeostasis by restoring the expression of genes associated with the regulation of cell proliferation and genomic integrity, such as transcription factors E2F, MYC, and the G2M checkpoint.

As reported in the literature, the resolution of inflammation is a dynamic process that occurs in distinct phases, starting from the down-regulation of pro-inflammatory genes and ending with the restoration of tissue homeostasis [48,49]. In accordance with this, the expression of genes related to homeostasis and cell proliferation remains lower also in the four-hour M-PS-treated samples compared to controls, while the expression of inflammatory genes returns to basal levels. The presence of these partially down-regulated genes could be viewed as a protective mechanism to prevent advancing through the cell cycle when cell homeostasis has not yet been completely restored. This finding suggests that a longer treatment might be necessary to fully reach cellular homeostasis, as already seen in our previous works where the anti-inflammatory potential was analysed as morphological and phenotypic recovery from developmental delay due to the copper sulphate treatment [9,11].

To validate gene expression results in vivo, we analysed the apoptosis process and the total antioxidant capacity (TAC). The significant decrease in cell death was in line with the reduction in apoptosis and p53 gene markers reported in the transcriptomic analysis, while TAC results confirmed that M-PS, and to a larger extent therapeutic thermal muds, exerted a significant antioxidant activity, leading to a reduction in ROS levels, as previously demonstrated with a different experimental approach [11].

Finally, it is important to note that, although in this paper we used polysaccharides extracted from a single mud sample, the chemical composition and reproducibility of M-PS effects can be considered consistent across different mud samples or batches. Indeed, in a recent paper, we demonstrated that M-PS extracted from six mature muds, produced by different spas of the Euganean Thermal District and in which mud maturation was performed at different temperatures, exhibit similar anti-inflammatory potential when analysed with the zebrafish model, despite changes in cyanobacterial population composition. This result is likely attributable to the chemical homogeneity of the molecules, both in terms of polysaccharide chemical structure and monosaccharide composition, which was highlighted by our chemical analyses [9].

## 5. Conclusions

Altogether, the findings described in this study suggest that M-PS, and Euganean thermal mud in general, play a key role in immune modulation, facilitating the resolution of inflammation and restoring tissue homeostasis.

These results lay the groundwork for possible future research on human subjects to further explore the molecular effects of peloid applications.

## Data Availability

Data contained within the article.

## Supplementary Materials

The following supporting information can be downloaded at: https://www.mdpi.com/article/10.3390/antiox14070878/s1, Figure S1: Schematic representation of the treatments used for the RNA sequencing (A) and for the RT-qPCR for the validation of the RNA sequencing results (B). Image created with Biorender.com. Figure S2: Schematic representation of image processing performed with the ImageJ software for the measurement of RawIndDen. Image created with Biorender.com. Figure S3: 11 clusters identified by the DEG pseudo-temporal pattern analysis. Table S1: Primer pairs used for RT-qPCR. Table S2: List of the up-regulated pathways identified with the Gene set enrichment analysis (GSEA). The tables report sample cluster, description of the hallmark sets, *p*-value and p.adjust, list and number of genes differentially expressed. Table S3: List of the down-regulated pathways identified with the Gene set enrichment analysis (GSEA). The tables report sample cluster, description of the hallmark sets, *p*-value and p.adjust, list and number of genes differentially expressed. Table S4: Exact adjusted P value resulted from one-way ANOVA followed by Tukey’s multiple comparison test of graphs of Figure 6.
