# Supplementary material for: Anti-Inflammatory Pathways Modulated by Microbial Polysaccharides from Euganean Thermal Muds in Zebrafish
Source: Antioxidants (Basel). 2025 Jul 17;14(7):878. doi: 10.3390/antiox14070878 (PMC12292280; doi:10.3390/antiox14070878)
Supplement: Supplementary file 1 [file antioxidants-14-00878-s001.zip › antioxidants-3713515-supplementary.pdf]

Anti-Inflammatory Pathways Modulated by Microbial Polysaccharides from Euganean Thermal Muds in Zebrafish

Micol Caichiolo [1,2,†], Raffaella Margherita Zampieri [1,†,‡,§], Francesca Terrin [1], Annachiara Tesoriere [1], Fabrizio Caldara [2], Nicoletta La Rocca [1], Paolo Martini [3,*] and Luisa Dalla Valle [1,*]

[1] Department of Biology, University of Padova, Via U. Bassi 58/b, 35131 Padova, Italy; micol.caichiolo@phd.unipd.it (M.C.); raffaellamargherita.zampieri@unifi.it (R.M.Z.); francesca.terrin@phd.unipd.it (F.T.); annachiara.tesoriere@phd.unipd.it (A.T.); nicoletta.larocca@unipd.it (N.L.R.)

[2] Pietro d'Abano Thermal Studies Center, Via Jappelli 5, 35031 Padova, Italy; fabrizio.caldara@centrostuditermali.org

[3] Department of Molecular and Translational Medicine, University of Brescia, Viale Europa 11, 25123 Brescia, Italy

[*] Correspondence: paolo.martini@unibs.it (P.M.); luisa.dallavalle@unipd.it (L.D.V.)

[†] These authors contributed equally to the work.

[‡] Present address: Department of Agriculture, Food, Environment and Forestry, University of Florence, Via San Bonaventura 13, 50145 Florence, Italy.

[§] Present address: Research Institute on Terrestrial Ecosystems (IRET), National Research Council (CNR), Via Madonna del Piano 10, 50019 Firenze, Italy.

A

2 h		2 h	2 h	Treatment name
FW	Rinse with FW: 4 x 15 min	FW		CTRL
FW		M-PS exposure		M-PS
$CuSO_4$ exposure		FW		INF
$CuSO_4$ exposure		M-PS exposure		INF + 4 h
$CuSO_4$ exposure		M-PS exposure	INF + 2 h	

B

2.5 h		4 h	2 h	Treatment name
FW	Rinse with FW: 4 x 15 min	FW		CTRL
$CuSO_4$ exposure		FW		INF
$CuSO_4$ exposure		M-PS exposure		INF + 6 h
$CuSO_4$ exposure		M-PS exposure	INF + 4 h	

Figure S1. Schematic representation of the treatments used for the RNA sequencing (A) and for the RT-qPCR for the validation of the RNA sequencing results (B). Image created with Biorender.com.

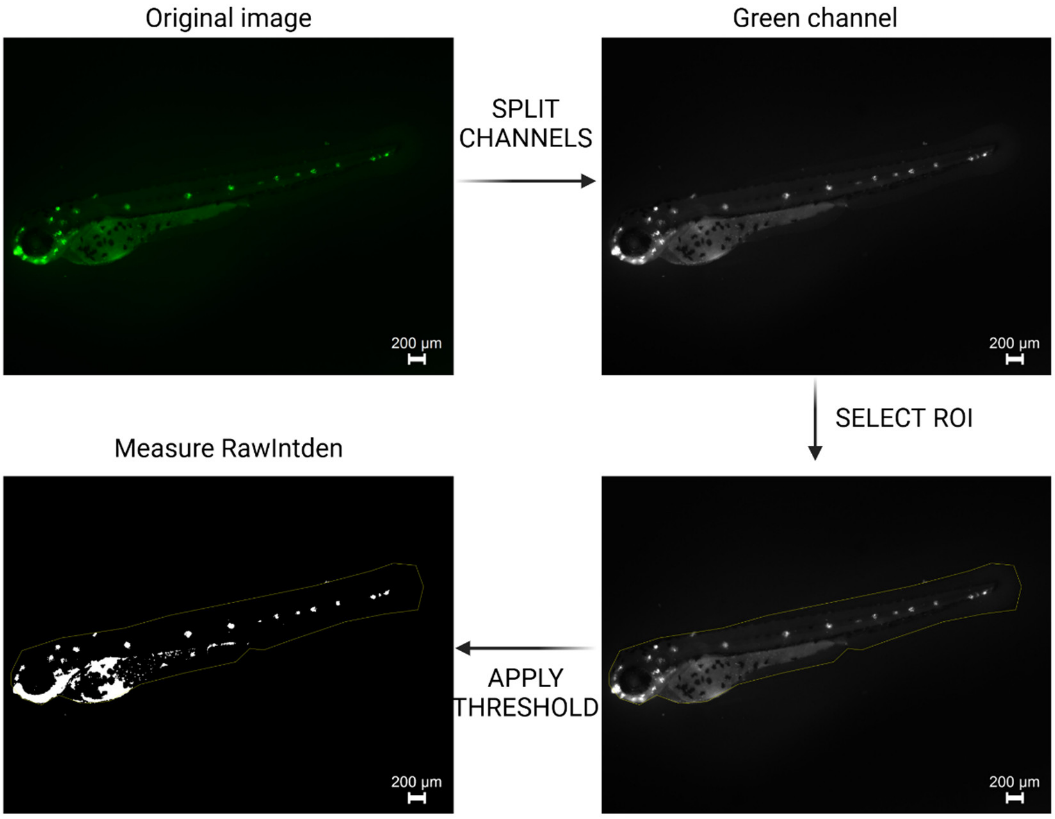

Figure S2. Schematic representation of image processing performed with the ImageJ software for the measurment of RawIndDen. Image created with Biorender.com.

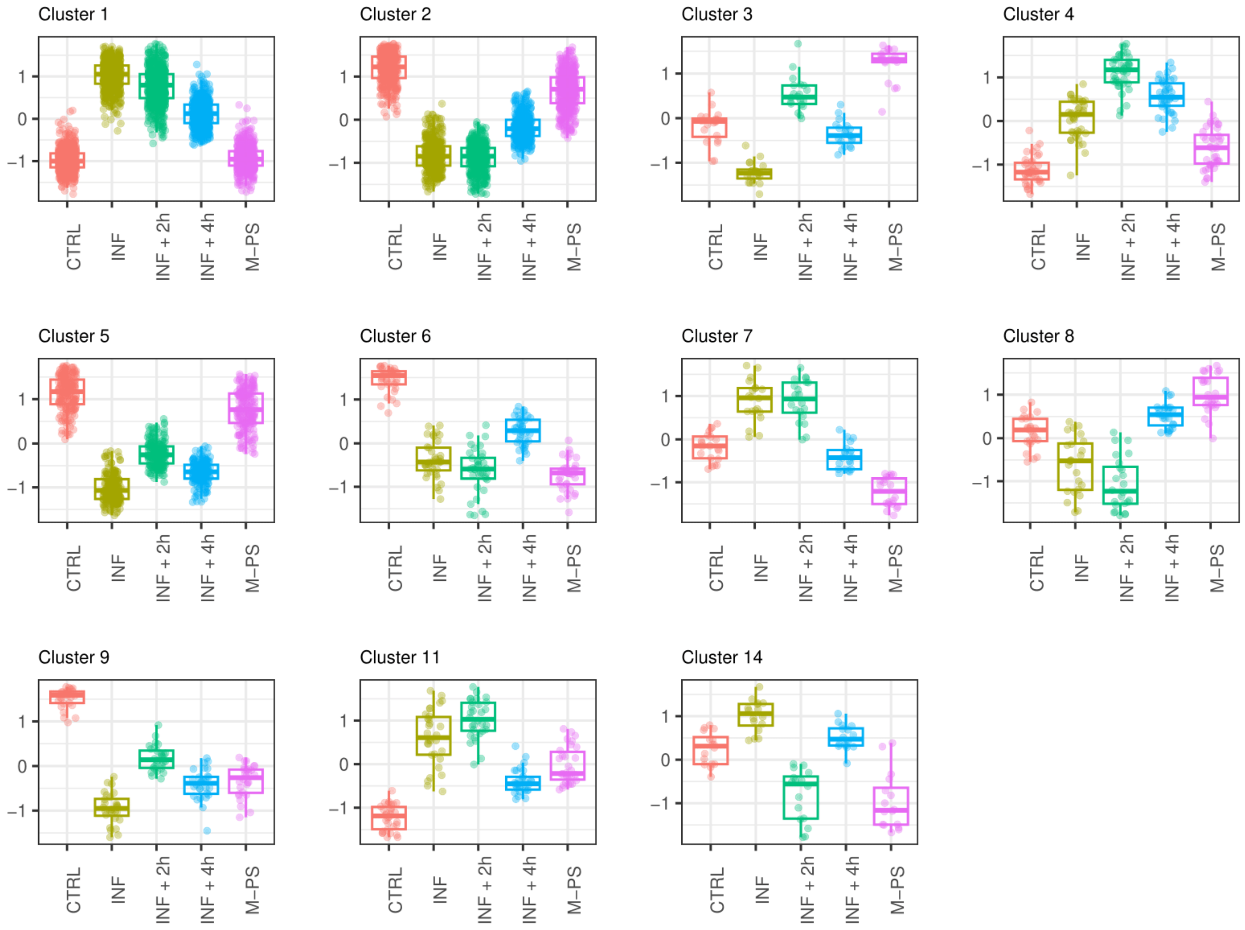

Figure S3. 11 clusters identified by the DEG pseudo-temporal pattern analysis.

Table S1: Primer pairs used for RT-qPCR.

Gene name	GeneBank accession No	Primer sequences (5′ - 3′)
gapdh	NM_001115114	*Fw*: GTGGAGTCTACTGGTGTCTTC<br>*Rv*: GTGCAGGAGGCATTGCTTAC
mmp13	NM_001290479	*Fw*: ATGGTGCAAGGCTATCCCAAGAGT<br>*Rv*: GCCTGTTGTTGGAGCCAAACTCAA
hsp70.3	NM_131397	*Fw*: GCAACGTGCTGATCTTTGACC<br>*Rv*: CAGATGAGTGTCTCCAGCG
socs3a	DQ333315.1	*Fw*: GGAAGACAAGAGCCGAGACT<br>*Rv*: GCGATACACACCAAACCCTG
ucp2	NM_131176	*Fw*: CACTGGACACCGCAAAAGTT<br>*Rv*: CGTACCAAAGACCCCTCGAT
fosab	NM_205569	*Fw*: CGTGAACGAAACAAGATGGCTG<br>*Rv*: ATTTTTCATCCTCAAGCTGGTCAG
jumba	NM_213556	*Fw*: CTCCGCTCCGAAATCGACT<br>*Rv*: TCCTCTGTGATGCTCCGAC

Table S2. List of the up-regulated pathways identified with the Gene set enrichment analysis (GSEA). The tables report sample cluster, description of the hallmark sets, *p*-value and p.adjust, list and number of genes differentially expressed.

Cluster	Description	*p*-value	p.adjust	geneID	Count
INF vs. CTRL	TNFA_SIGNALING_VIA_NFKB	3,68E-11	1,80E-09	pfkfb3/nfkbiaa/atf3/mcl1a/ptgs2b/fosl1a/dusp5/stat5a/btg2/socs3a/sgk1/socs3b/btg1/klf6a/fosab/irs2a/fosl2/cebpb/nfe2l2a/serpine1/sdc4/slc16a6b/bcl6aa/junba/abca1a/sqstm1/hbegfa/serpinb14/f3a/zgc:158343/zc3h12ab/ets2/il6st	33
INF vs. CTRL	P53_PATHWAY	2,73E-08	6,69E-07	abat/atf3/caspa/mknk2b/trib3/btg2/sesn1/s100a10b/btg1/hmox1a/itgb4/fosab/mdm2/tp53/cebpa/txnipa/prkab1a/tp63/rrad/zgc:110425/ddit3/st14a/abcc5/baiap2a/perp/vdrb/hbegfa/kif13ba/zgc:158343/abhd4	30
INF vs. CTRL	APOPTOSIS	3,30E-06	5,40E-05	casp9/atf3/caspa/mcl1a/clu/slc20a1b/lmna/krt18a.1/btg2/tspo/anxa1a/hmox1a/txnipa/h1-0/bmf2/gpx3/etf1b/ddit3/timp2a/cth/sqstm1/zgc:158343/si:dkey-11f4.20	23
INF vs. CTRL	IL6_JAK_STAT3_SIGNALING	3,83E-05	0,000469364	tnfrsf21/stat1a/ptpn1/stat3/socs3a/socs3b/hmox1a/stat2/lifrb/pim1/il6st	11
INF vs. CTRL	INTERFERON_GAMMA_RESPONSE	0,0003061	0,002999628	nfkbiaa/stat1a/ptpn1/caspa/ptgs2b/ifi27.1/mvp/stat3/socs3a/socs3b/btg1/stat2/txnipa/mt2/cfb/bpgm/pim1	17
INF vs. CTRL	APICAL_JUNCTION	0,0008857	0,00701315	myl9b/cldnb/cldn3d/cldn11a/itgb4/itga9/cnn2/mdka/cldn7a/cldn8.2/mmp9/cldne/baiap2a/si:ch73-22o12.1/itga2.2/pik3cb/zyx/ctnnd1/adam15/actn4/ctnna1/cdh1	22
INF vs. CTRL	COMPLEMENT	0,0010019	0,00701315	pla2g7/casp9/ctsla/caspa/clu/c3a.1/dusp5/calm1a/mt2/cebpb/c3a.3/cfb/f5/hsp70l/serpine1/timp2a/ppp4cb/scg3/f3a/pim1	20
INF vs. CTRL	UV_RESPONSE_UP	0,0042712	0,02616113	nfkbiaa/atf3/ctsla/olfm1b/stard3/btg2/btg1/hmox1a/fosab/epcam/dnajb1b/gpx3/rrad/h2ax1/cdc34a/junba/sqstm1/il6st	18
INF vs. CTRL	HEME_METABOLISM	0,0120748	0,065740691	optn/sdcbp2/c3a.1/rad23aa/bmp2k/btg2/tspo/smox/h1-0/mkrn1/riok3/ucp2/isca1/c3a.3/lrp10/bpgm/bcam/mpp1	18
INF vs. CTRL	COAGULATION	0,0200203	0,098099243	casp9/ctsla/clu/mmp13a/c3a.1/anxa1a/fgg/mmp9/c3a.3/cfb/serpine1/itga2.2/f3a	13
INF + **2 h** vs. CTRL	TNFA_SIGNALING_VIA_NFKB	3,63E-13	1,78E-11	pfkfb3/atf3/mcl1a/gadd45bb/fosl1a/dusp5/btg2/socs3a/sgk1/socs3b/klf6a/ldlra/fosab/smad3a/tsc22d1/fosl2/cebpb/nfe2l2a/serpine1/sdc4/slc16a6b/tnf	32

				rsf9a/tiparp/gch1/junba/abca1a/sqstm1/serpinb14/zc3h12ab/vegfaa/ets2/il6st	
INF + **2 h** vs. CTRL	IL6_JAK_STAT3_SIGNALING	4,59E-06	0,000112482	tnfrsf21/ptpn1/stat3/socs3a/socs3b/hmox1a/stat2/lepr/il13ra1/pim1/il6st	11
INF + **2 h** vs. CTRL	P53_PATHWAY	3,73E-05	0,000609024	abat/atf3/caspa/mknk2b/btg2/s100a10b/hmox1a/itgb4/fosab/txnipa/tsc22d1/xpc/ddb2/tp63/rrad/zgc:110425/st14a/abcc5/perp/vdrb/abhd4	21
INF + **2 h** vs. CTRL	COMPLEMENT	0,0001557	0,00190788	pla2g7/casp9/ctsla/caspa/clu/cblb/dusp5/calm1a/mt2/cebpb/cfb/f5/hsp70l/serpine1/timp2a/gpd2/ctss2.1/scg3/pim1	19
INF + **2 h** vs. CTRL	APOPTOSIS	0,0002048	0,002006601	casp9/atf3/caspa/mcl1a/clu/slc20a1b/lmna/gadd45bb/krt18a.1/btg2/anxa1a/hmox1a/txnipa/h1-0/timp2a/gch1/sqstm1	17
INF + **2 h** vs. CTRL	IL2_STAT5_SIGNALING	0,0039332	0,032121094	tnfrsf21/mapkapk2a/odc1/gadd45bb/klf6a/capn3b/alcamb/cdcp1a/tnfrsf9a/myo1cb/ahnak/slc1a5/pim1/hk2/irf6	15
INF + **2 h** vs. CTRL	UV_RESPONSE_UP	0,0063396	0,043832305	atf3/ctsla/stard3/btg2/hmox1a/fosab/epcam/dnajb1b/rrad/h2ax1/gch1/junba/sqstm1/cyp1a/il6st	15
INF + **2 h** vs. CTRL	INTERFERON_GAMMA_RESPONSE	0,0071563	0,043832305	ptpn1/caspa/mvp/stat3/socs3a/socs3b/stat2/txnipa/mt2/cfb/gch1/pim1	12
INF + **4 h** vs. CTRL	APOPTOSIS	0,0003814	0,015254214	lmna/krt18a.1/tspo/anxa1a/hmox1a/h1-0/timp2a/sqstm1	8
INF + **4 h** vs. CTRL	UV_RESPONSE_UP	0,0041475	0,082949968	ctsla/hmox1a/h2ax1/junba/sqstm1/cyp1a/il6st	7

Table S3. List of the down-regulated pathways identified with the Gene set enrichment analysis (GSEA). The tables report sample cluster, description of the hallmark sets, p-value and p.adjust, list and number of genes differentially expressed.

**Cluster**	**Description**	**p-value**	**p.adjust**	**geneID**	**Count**
INF vs. CTRL	E2F_TARGETS	7,65E-12	3,60E-10	rpa3/rpa1/stmn1a/kif4/ezh2/nop56/rfc2/ranbp1/pola2/gins4/msh2/rnaseh2a/mcm5/mcm3/ing3/rpa2/tubb5/nasp/mcm4/dck/dlgap5/pcna/rfc3/usp1/srsf2a/mcm6/hells/lig1/ube2t/dek/mms22l/orc6/pold3/lyar/cdc20/asf1ba/mcm2/chek1/dut	39
INF vs. CTRL	MYC_TARGETS_V1	3,14E-08	7,38E-07	hnrnpub/pole3/eif3ja/nop56/rrm1/ranbp1/ncbp2/ndufab1b/hnrnpua/dhx15/hdac1/mcm5/psmd8/rpl34/psmc6/mcm4/tyms/vdac1/mrpl23/cnbpa/eif2	34

				s2/hnrnpc/pcna/hspe1/usp1/srsf2a/mcm6/eif3ba/dek/bub3/cdc20/mcm2/rpl14/dut	
INF vs. CTRL	G2M_CHECKPOINT	8,31E-06	0,000130167	stmn1a/hnrnpub/kif4/ezh2/pola2/hnrnpua/dkc1/mcm5/orc5/mcm3/rpa2/nasp/prpf4bb/atrx/arid4a/lig3/srsf2a/mcm6/chaf1a/top1a/orc6/bub3/srsf10b/incenp/cdc20/mcm2/slc12a2/chek1	28
INF vs. CTRL	OXIDATIVE_PHOSPHORYLATION	0,0001376	0,0016173	atp5fa1/nqo1/atp5pb/gpia/ndufab1b/mdh1aa/atp5mc3a/hsd17b10/idh1/cox4i1/slc25a11/bckdha/mdh2/lrpprc/vdac1/timm13/cpt1aa/timm10/oxa1l/atp5f1b/atp5mea/oat/mpc1/mrpl15/atp6v0e1/cs	26
INF vs. CTRL	MTORC1_SIGNALING	0,0003039	0,002856203	rpa1/elovl6/stip1/gpia/cacybp/psmd13/idh1/ppa1b/psmc6/mcm4/arpc5la/psmd12/nmt1a/eif2s2/hspe1/rpn1/tuba8l3/serpinh1a/zgc:92744/calr/psma3/mcm2/hmgcs1/uchl5	24
INF vs. CTRL	DNA_REPAIR	0,0016265	0,012740712	rpa3/fen1/hcls1/rfc2/ncbp2/pola2/gtf2f1/rpa2/tyms/pola1/ralaa/pcna/arl6ip1/rfc3/lig1/pold3/nme3/dut	18
INF + **2 h** vs. CTRL	E2F_TARGETS	6,64E-25	3,06E-23	rpa3/rpa1/stmn1a/kif4/ubr7/cse1l/ezh2/cdc25b/rfc2/ranbp1/pola2/tubg1/gins4/msh2/rnaseh2a/mcm5/dscc1/mcm3/pold1/dnmt1/mybl2b/gins3/nasp/mcm4/asf1bb/dck/dlgap5/pcna/suv39h1b/rfc3/usp1/hmgb3a/mcm6/hells/smc1al/pole/lig1/dek/mms22l/orc6/pold3/pold2/lyar/eed/nup153/cdc20/asf1ba/mcm2/psip1a/chek1/dut/zgc:86839	52
INF + **2 h** vs. CTRL	G2M_CHECKPOINT	1,01E-10	2,31E-09	gins2/stmn1a/hnrnpub/kif4/ezh2/cdc25b/rbm14a/pola2/hnrnpua/smarcc1a/mcm5/casp8ap2/mcm3/mybl2b/nasp/prpf4bb/atrx/arid4a/suv39h1b/hmgb3a/mcm6/smc1al/pole/chaf1a/top1a/orc6/bub3/prmt5/srsf10b/incenp/cdc20/mcm2/chek1/zgc:86839	34
INF + **2 h** vs. CTRL	MYC_TARGETS_V1	1,80E-06	2,76E-05	psmd1/cbx3a/hnrnpub/pole3/eif3ja/rrm1/ranbp1/hnrnpua/hdac1/smarcc1a/mcm5/psmd8/psmc6/mcm4/rfc4/tyms/mrpl23/cnbpa/eif2s2/pcna/usp1/mcm6/dek/bub3/pold2/cdc20/mcm2/dut	28
INF + **2 h** vs. CTRL	DNA_REPAIR	0,0008536	0,009816056	rpa3/fen1/hcls1/rfc2/pola2/pold1/gtf2f1/rfc4/tyms/pcna/arl6ip1/rfc3/gtf2h5/lig1/pold3/gpx4b/dut	17
INF + **4 h** vs. CTRL	G2M_CHECKPOINT	0,0001519	0,003075789	hnrnpub/kif4/hnrnpua/dkc1/mybl2b/nasp/lig3/top1a/srsf10b/mcm2	10

INF + **4 h** vs. CTRL	E2F_TARGETS	0,0001809	0,003075789	kif4/rfc2/rnaseh2a/mybl2b/nasp/mcm4/pcna/lig1/mcm2/dut	10
INF + **4 h** vs. CTRL	MYC_TARGETS_V1	0,0012096	0,013709261	hnrnpub/rrm1/npm1a/hnrnpua/hdac1/mcm4/pcna/mcm2/dut	9
INF + **4 h** vs. CTRL	UV_RESPONSE_UP	0,0025537	0,021706082	hnrnpub/stip1/psmc3/hnrnpua/rpn1/tuba8l3/cdo1	7
M-PS vs CTRL	OXIDATIVE_PHOSPHORYLATION	0,0095735	0,038294135	mpc1/cs	2

Table S4: Exact adjusted P value resulted from one-way ANOVA followed by Tukey's multiple comparison test of graphs of **Figure 6**.

**Tukey's multiple comparisons test**	***mmp13***	***hsp70.3***	***socs3a***	***ucp2***	***fosab***	***junba***
CTRL vs. INF	<0,0001	<0,0001	0,0012	0,0002	0,0473	0,0246
CTRL vs. INF + **4 h**	<0,0001	<0,0001	0,2845	0,0370	0,9283	0,5802
CTRL vs. INF + **6 h**	<0,0001	0,3789	0,9946	0,1338	0,9410	0,1729
INF vs. INF + **4 h**	0,9600	0,4508	0,0291	0,0336	0,1310	0,2155
INF vs. INF + **6 h**	0,0018	<0,0001	0,0017	0,0089	0,0177	0,0006
INF + **4 h** vs. INF +**6 h**	0,0040	0,0007	0,3943	0,8705	0,6562	0,0191
